# Exploring Beyond the Cyclical Dynamics of Water, Sanitation, and Hygiene With Coexisting Forms of Malnutrition Among Children Under 5 in Pakistan

**DOI:** 10.1155/ijpe/6378814

**Published:** 2026-09-09

**Authors:** Bushra Ashar, Haji Abdul Rehman Akhter, F. N. U. Sumia, Izzan Ahmed Usmani, Muhammad Hakim Khan, Kamil Ahmad Kamil, Asif Khaliq

**Affiliations:** ^1^ Department of General Surgery, Karachi Medical and Dental College, Karachi, Sindh, Pakistan; ^2^ Department of Surgery, CMH Multan Institute of Medical Sciences, Multan, Punjab, Pakistan; ^3^ Department of Medicine, Peoples University of Medical and Health Sciences for Women, Nawabshah, Sindh, Pakistan, duhs.edu.pk; ^4^ Department of Medicine, Dow International Medical College (DIMC), Karachi, Sindh, Pakistan, duhs.edu.pk; ^5^ Department of Medicine, Kandahar University, Kandahar, Afghanistan, kdru.edu.af; ^6^ School of Public Health and Social Work, Queensland University of Technology (QUT), Brisbane, Australia, qut.edu.au

**Keywords:** coexisting forms of malnutrition, diarrhea, hygiene, maternal education, Pakistan, sanitation, water

## Abstract

**Background:**

Coexisting forms of malnutrition (CFM) represent a critical global health priority, particularly among children under 5, owing to their severe consequences and elevated mortality risk compared to standalone forms of malnutrition. This study was aimed at assessing the relationship between household water, sanitation, and hygiene (WASH) conditions and CFM among children under 5 years in Pakistan.

**Subjects and Method:**

This cross‐sectional study utilized data from the last two waves of the Pakistan Demographic and Health Survey (PDHS) conducted in 2012–2013 and 2017–2018. From these datasets, data for a total of 6874 children aged between 0 and 59 months were included for analysis. Children who were non‐de jure residents, deceased at the time of survey, or had missing or flagged anthropometric data (outliers) were excluded. The data was analyzed using bivariate and multivariate logistic regression models. The four CFM outcomes examined were coexistence of underweight with wasting (CUW), coexistence of underweight with stunting (CUS), coexistence of underweight, wasting, and stunting (CUWS), and coexistence of stunting with overweight/obesity (CSO), each defined using WHO anthropometric cut‐off values (*z*‐score ≤ −2 SD for undernutrition indices and ≥ +2 SD for overweight/obesity).

**Results:**

Key findings revealed three distinct WASH‐nutrition patterns. First, improved drinking water access was strongly associated with concurrent under‐ and overnutrition. CUW and CSO had lower odds by 85% and 59%, respectively. Second, infection prevention played a significant role. Children without diarrhea and fever in the last 14 days were associated with lower odds of CSO by 38%–45%, highlighting the protective role of infection prevention. Third, socioeconomic and maternal factors showed divergent effects. Children of highly educated mothers had 72% lower odds of CUS compared to uneducated mothers. Both maternal and paternal employment had a protective role in various forms of CFM. Additionally, children from the richest households demonstrated decreased odds of CUW (OR: 0.13, CI: 0.05–0.34) but increased odds of CSO (OR: 2.43, CI: 1.46–4.03), signifying a nutritional transition associated with affluence.

**Conclusion:**

This study is among the first to demonstrate that distinct WASH indicators differentially shape coexisting malnutrition patterns in Pakistani children under 5. Improved drinking water access mitigates concurrent undernutrition. Higher household wealth paradoxically elevates the risk of stunting–overweight coexistence, underscoring the dual burden of malnutrition in transitioning economies. These findings call for integrated WASH‐nutrition policies with equity‐sensitive approaches to accelerate Pakistan′s progress toward SDGs 2 and 6.

## 1. Introduction

Combating malnutrition in all its forms is one of the greatest global health challenges influenced by factors such as economic growth and urbanization [1, 2]. The global nutrition landscape presents an increasingly complex paradigm [3]. According to the global estimates of 2022, around 230.1 million children under 5 years of age are affected by various forms of malnutrition. However, more than half of the cases of pediatric malnutrition, 125.9 million (~54.7%), are from Asian countries [4]. Malnutrition manifests in both standalone and coexisting forms [5]. The standalone forms of malnutrition (SFM) include stunting, wasting, underweight, overweight, obesity, and micronutrient deficiencies, whereas the coexisting forms of malnutrition (CFM) represent the simultaneous presence of undernutrition alongside overnutrition [5, 6]. Each CFM subtype in our study represented a clinically distinct phenotype. Coexistence of underweight with wasting (CUW) signals an acute nutritional crisis. Coexistence of underweight with stunting (CUS) and coexistence of underweight, wasting, and stunting (CUWS) reflect compounded chronic and acute deficits associated with markedly elevated mortality risk [7, 8]. Coexistence of stunting with overweight/obesity (CSO) reflects the individual‐level double burden of malnutrition (DBM), where stunting and excess adiposity coexist in the same child, a pattern increasingly prevalent in economies undergoing nutritional transition [3, 5]. Importantly, CSO differs from the household‐level double burden (where undernutrition and overnutrition affect different household members). CSO represents an individual‐level paradox driven by early life undernutrition alongside later energy surplus [9].

Worldwide, CFM affects more than half of malnourished children, who have more than 10 times higher risk of death, whereas children with SFM have a twofold higher risk of death compared to a normal healthy child [10, 11]. In young children, around half of the cases of malnutrition are caused by fecal‐transmitted infections (FTIs), including diarrheal diseases such as hepatitis A and cholera, vector‐borne infections (e.g., malaria and typhoid), and soil‐transmitted helminths (STHs) such as hookworm and schistosomiasis [10]. Such infections can lead to anemia, reduced physical development, and inhibited cognitive development [11]. Furthermore, exposure to FTIs, including diarrheal disease and environmental enteropathy (environmental enteric dysfunction [EED]) secondary to intestinal worms, has a synergistic relationship with pediatric undernutrition [12]. Most of the nutrition‐related factors, such as low birth weight, stunting, and wasting (defined by the World Health Organization [WHO] *z*‐score cut‐offs; see Methodology), are interlinked to a lack of access to water, sanitation, and hygiene (WASH) [13]. Worldwide, over five billion people around the world lack access to safe drinking water and clean sanitation services [14]. Moreover, lack of sanitation and hygiene is estimated to cause over a million deaths each year, specifically among young children [15, 16]. Children living in most of the developing countries are constantly facing the issues of poor WASH, leading to diarrhea and other FTIs, which contribute to pediatric malnutrition [17, 18]. Pakistan′s progress in child malnutrition has not been encouraging. A study conducted by WHO in 2022 revealed that Pakistan stands third among the Top 10 countries for the highest number of deaths for children under 5 in 2020 [19]. The National Nutrition Survey (NNS) of Pakistan conducted in 2018 showed the highest prevalence of pediatric wasting compared with the former NNS and other health and nutrition surveys. The prevalence of pediatric wasting reported in the 2018 NNS was 17.7%; however, the former NNS conducted in 2001 and 2011 reported 13% and 15.1% prevalence of wasting among children below 5 years [20, 21]. Thus, the burden of pediatric wasting in Pakistan is continuously increasing and reaching an alarming threshold. Similarly, the prevalence of stunting in Pakistan, which is a chronic form of malnutrition, remains stagnant at around 40% over the last 4–5 decades [20, 22]. Moreover, an estimated 70% of Pakistani households consume contaminated water, and 56.1% have drinking water contaminated with coliforms [21], driving FTI‐related child mortality. In Pakistan, the progress toward Sustainable Development Goals (SDGs) related to WASH and nutrition remains slow, necessitating increased resource allocation for these programs [23].

The evidence regarding the association of WASH conditions and malnutrition has been supported by various observational and experimental studies, but the relationship between different types of WASH conditions and various forms of CFM has not been explored. There has been increasing global recognition that all types of malnutrition need to be addressed [9, 24]. Target 2.2 of the SDGs is to “end malnutrition in all its forms” [9, 24], and the Lancet Commission on the global syndemic of obesity, undernutrition, and climate change highlights “the need to tackle these interconnected problems simultaneously” [25]. To the best of our knowledge, this is the first nationally representative, dual‐survey study from Pakistan to address critical gaps in understanding the relationship between different types of WASH indicators (improved and unimproved), and all four established CFM subtypes (CUW, CUS, CUWS, and CSO) among children under 5 years using two nationally representative waves of PDHS 2012–2013 and 2017–2018. The findings will inform evidence‐based interventions and policy recommendations for integrated WASH‐nutrition programs.

## 2. Methodology

### 2.1. Study Design and Setting

This is an analytical cross‐sectional study using secondary data from the PDHS that are nationally representative and relevant to this study because they capture child anthropometry, household WASH conditions, and sociodemographic covariates at the population level [26].

Pakistan has participated in four DHS since the time of its establishment. In this study, datasets from the third wave (2012–2013) and fourth wave (2017–2018) of PDHS were used to assess the relationship between WASH conditions and CFM among children under 5 years of age in Pakistan [27, 28]. However, the datasets of the first two PDHS conducted in 1990–1991 and 2006–2007 were excluded, as earlier waves lacked the nutritional and anthropometric variables required for the present analysis.

### 2.2. Study Participants and Their Eligibility

In each PDHS, women between the ages of 15 and 49 years were approached via a multistage stratified cluster sampling method. This study specifically collected data from those women who had a child aged between 0 and 59 months. Children with valid and complete anthropometric measurements were included in this study. However, data from non‐de jure residents, deceased children, and children either with missing anthropometry or anthropometric red flags/outliers were excluded. The WHO has described different ranges of anthropometric outliers for each nutritional category. A cut‐off value exceeding ±6.00 SD for length/height for age (LAZ/HAZ), ±5.00 SD for weight for length/height (WHZ), and ±6.00 SD for weight for age (WAZ) was considered an outlier [5, 29, 30].

### 2.3. Sample Size and Sampling Method

DHS sample designs are two‐stage probability samples drawn from an existing sample frame, generally the most recent census frame. In the first stage, primary sampling units (PSUs), usually census enumeration blocks (EBs), are selected with probability proportional to size (PPS) within each stratum. The PSU forms the survey cluster, a fixed take of households per cluster of about 25–30 households. In the second stage, a household listing is conducted in each selected EB, and then a fixed number of households is chosen using equal probability systematic sampling. The principal objective of multistage sampling is to reduce sampling errors. Typically, DHS samples are stratified by geographic region and by urban/rural areas within each region [28]. The Pakistan Bureau of Statistics (PBS) supported the sample design of the PDHS and worked in close coordination with the National Institute of Population Studies (NIPS). The survey estimates are also representative for the four provinces of Punjab, Sindh, Khyber Pakhtunkhwa, and Baluchistan; for two regions including Azad Jammu and Kashmir (AJK) and Gilgit Baltistan (GB); for Islamabad Capital Territory (ICT); and for Federally Administered Tribal Areas (FATA). In total, there were 13 second‐level survey domains, 16 sampling strata were created, and the number of EBs in 2012–2013 was 500, which increased to 580 in 2017–2018. The survey team multiplied the number of EBs by the fixed number “28,” thus producing a sample size of 14,000 and 16,240 [27, 28].

The final analytic sample comprised *n* = 6874 children under 5 years (2917 from 2012–2013 and 3957 from 2017–2018) after applying eligibility criteria. Exclusion details by dataset, including counts of non‐de jure residents, deceased children, and anthropometric outliers removed from each wave, are summarized in Table 1.

**Table 1:** Data of the participants from each dataset included in the study.

Pakistan demographic and health survey			
	2012–2013	2017–2018	Total
Total sample size (exclude: Households without children)	11,763 (7806)	12,708 (8209)	24,471 (16,015)
Household having children 0–5 years (exclude: Non‐de jure residents)	3957 (172)	4499 (159)	8456 (331)
Data of de jure residents (exclude: Children with incomplete and/or missing anthropometric outliers)	3785 (868)	4340 (383)	8125 (1251)
Sample included	2917	3957	6874

### 2.4. Conceptual Framework

**Figure 1:**
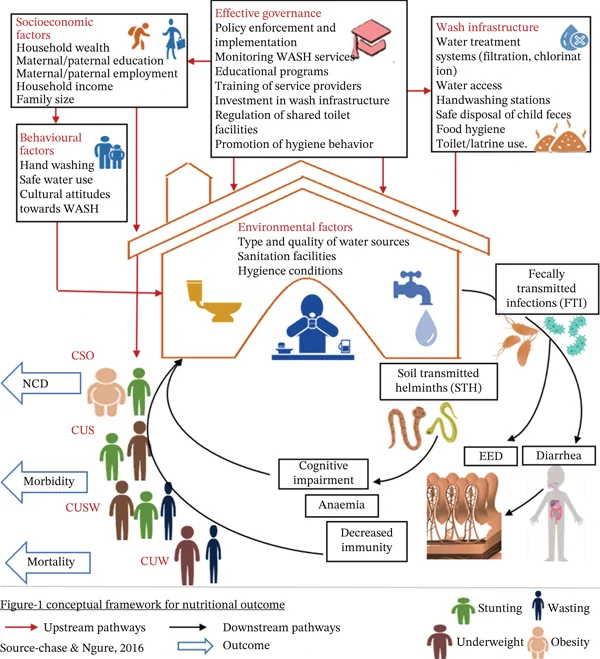
Conceptual framework showing interlinking pathways between WASH and nutritional outcomes. Pictorial representation of the conceptual framework of WASH facilities and CFM; black arrows show the direct association leading to a vicious cycle, whereas the red arrows show the indirect, interconnected pathways that are multisectoral and complex. Blue arrows are the ultimate outcomes of coexisting forms of malnutrition. SO, coexisting stunting and obesity; CUS, coexistence of underweight with stunting; CUWS, coexistence of underweight, wasting, and stunting; CUW, coexistence of underweight with wasting; NCD, noncommunicable diseases; EED, environmental enteric dysfunction.

WASH influences child nutritional outcomes through two interconnected pathways, illustrated in Figure 1 [31, 32].

#### 2.4.1. Downstream Pathways/Cyclical Pathways/Direct Pathways

Downstream (direct) pathways operate through fecal–oral transmission: Contaminated water and poor sanitation promote diarrheal disease, STHs, and EED, all of which impair nutrient absorption and drive the malnutrition‐infection vicious cycle [33, 34].

#### 2.4.2. Upstream Pathways/Interlinked Pathways/Indirect Pathways

Upstream (indirect) pathways encompass the broader socioeconomic, environmental, behavioral, and governance determinants, including poverty, maternal education, and infrastructure access, that shape household WASH conditions and hygiene practices and, in doing so, modulate the downstream infection risk. The full interplay of both pathway types is illustrated in Figure 1 [32].

### 2.5. Measurement of Exposure Variable

The primary exposure of this study was WASH practices in the household. The information related to the WASH variable was collected by a team of trained and experienced data collectors from the household questionnaire, which contained a set of categories for assessing the type of water and sanitation facilities provided.

Using the WHO/SDG core questions and indicators for monitoring WASH [35, 36], water source and sanitation data were each recoded into binary categories. For drinking water: Piped water, boreholes or tube wells, protected dug wells, protected springs, rainwater, and packaged or delivered water were classified as improved; unprotected dug wells, unprotected springs, and surface water were classified as unimproved. For sanitation: Flush/pour–flush toilets (connected to sewers, septic tanks, or pit latrines), pit latrines with slabs, and composting toilets were classified as improved; pit latrines without slabs, hanging toilets, and bucket toilets were classified as unimproved [36].

### 2.6. Measurement of Outcome Variables

The anthropometric data of children aged 0–59.9 months was collected by a team of trained and experienced anthropometrists. A team of two anthropometrists measured the height/length and weight of each child using a calibrated and standardized weighing scale and stadiometer. The data related to child weight (in kilograms), height/length (in centimeters), age (in months), gender (male/female), and measuring position (lying/standing) were then transferred to the WHO AnthroCal application for the calculation of *z*‐score (AnthroCal, 2006).

The *z*‐score value was used to assess the nutritional status of each child. Three different types of anthropometric indices, such as LAZ/HAZ, WAZ, and WLZ/WHZ, were used to measure the nutritional status of each child. Children classified below −2.00 SD on LAZ/HAZ, WAZ, or WLZ/WHZ were considered stunted, underweight, or wasted, respectively; those above +2.00 SD on WAZ or WLZ/WHZ were considered overweight/obese children [5, 30] (note: Detailed *z*‐score cut‐off ranges and AnthroCal classification criteria are provided in File S1 to reduce technical burden in the main text).

Following *z*‐score calculation and assigning nutritional status to each anthropometric index, computational analysis was performed to assess individual nutritional status. To perform the computational analysis, numeric codes were assigned to each different type of nutritional status. Based on the numeric code of each type of nutritional status, nine different types of nutritional status were identified, such as normal, stunting, wasting, underweight, CUS, CUW, CUWS, overweight/obesity, and CSO. Further detail regarding the coding and computational analysis of each type of nutritional status for each anthropometric index is provided in File S1.

### 2.7. Covariates

Some covariate variables were identified and included in the study that might influence the association of WASH practices with CFM. These were child factors, maternal factors, and household factors. Child factors included age (0–11, 12–23, 24–35, 36–47, and 48–59 months), sex (male and female), birth weight (low birth weight and normal birth weight), birth order (first childbirth and subsequent childbirth), presence of diarrhea (yes and no), fever (yes and no), and pneumonia (yes and no). Maternal factors included maternal age (15–19, 20–24, 25–29, 30–34, 35–39, 40–44 years, and 45–49 years), education level (no education, primary, secondary, and higher), employment status (yes and no), mode of delivery (cesarean section and vaginal delivery), BMI status (underweight, normal weight, overweight, and obese), and maternal smoking cigarettes (yes and no). Paternal factors included education level (no education, primary, secondary, and higher) and employment status (yes and no).

Household factors included type of place of residence (rural and urban), wealth index (poorest, poorer, middle, richer, and richest), and family size (small, medium, and large).

### 2.8. Statistical Analysis

#### 2.8.1. Data Preparation, Cleaning, and Management

The analysis began with a comprehensive data preparation process using the 2012–2013 and 2017–2018 PDHS datasets. After obtaining the datasets, we assessed their structure and completeness before excluding records of non‐de jure residents and deceased children. We recoded key variables, including water sources and sanitation facilities, into binary categories (improved vs. unimproved) according to WHO guidelines to simplify data for analysis. The anthropometric *z*‐scores were calculated using WHO AnthroCal software. However, the outliers were removed according to established criteria. Nutrition profile for each anthropometric index was determined using the *z*‐score cut‐off values; that is, any child with *z*‐score value between −1.99 and +1.99 SD was considered a normal healthy child, whereas children with deviant *z*‐score were classified either overnourished (*z*‐score value ≥ 2.00 SD) or undernourished (*z*‐score value ≤ 2.00 SD). New variables were created to represent each type of SFM and CFM. For each survey cycle, we merged relevant variables from household, women, and child datasets, then combined datasets of the two waves (n = 6874: 2917 from 2012 to 2013 and 3957 from 2017 to 2018) for subsequent statistical analyses.

#### 2.8.2. Descriptive Analysis

The descriptive analysis provided a comprehensive profile of the study population and key variables across both PDHS datasets (2012–2013 and 2017–2018). We calculated frequencies and percentages with 95% confidence intervals (CIs) for all categorical variables, including WASH indicators, malnutrition categories, and sociodemographic factors. The data from each dataset used in this study was initially analyzed descriptively, which involved assessing the percentage of each categorical variable. The continuous variables, like child age, maternal age, birth order, birth weight, BMI, and number of household members, were also categorized. A cumulative descriptive analysis was carried out by combining the two waves (2012–2013 and 2017–2018) datasets after each dataset had undergone a descriptive analysis.

#### 2.8.3. Inferential Analysis

Data preparation and variable recoding were performed in Microsoft Excel 2019; descriptive and inferential statistical analyses were conducted in SPSS Version 27 and Jamovi 2022 Version 2.3, respectively.

The CFM is the outcome variable of this study, which has four different types: CUW, CUS, CUWS, and CSO. The presence of either stunting or wasting, or both with underweight, is collectively referred to as coexisting forms of undernutrition, for which the reference category in our study was “underweight,” whereas the reference category for CSO was “stunting.” Before performing inferential statistics, four separate data files were created, one for each CFM type (CUW, CUS, CUWS, and CSO). Each contained the CFM type of interest and its corresponding reference category (underweight for coexisting undernutrition types; stunting for CSO). This was necessary because each logistic regression model required a distinct binary outcome with an appropriate reference group; a single merged dataset would not permit simultaneous modeling of all four CFM‐specific reference comparisons.

Inferentially, the data was analyzed by performing binomial and multivariate logistic regression. During binomial logistic regression, we examined the individual association between each study covariate and WASH conditions with different forms of malnutrition. We performed unordered logistic regression to examine each factor associated with each type of CFM by calculating unadjusted odds ratios with 95% CIs to quantify the strength of these associations with the outcome variables, details of which are provided in Table 2. In the multivariate logistic regression analysis, multiple logistic regression models were constructed to assess the relationship between WASH practices and various forms of malnutrition while controlling for potential covariates. The relationship of study outcome variables with all the study variables was analyzed in four steps simultaneously. Backward elimination was employed to build parsimonious models. Variables with *p* > 0.05 were sequentially removed until only significant predictors remained. This approach was chosen to reduce overfitting while retaining all a priori theoretically justified variables (WASH indicators, maternal education, and wealth) regardless of *p* value. Adjusted odds ratios with 95% CIs were calculated to quantify the relationships. The deviance, Akaike information criterion (AIC), Bayesian information criterion (BIC), and McFadden′s test were used to assess model stability, and variance inflation factors (VIFs) were calculated to check for multicollinearity. The statistical analysis was performed using Jamovi software.

**Table 2:** Characteristics of the study sample.

Variables	Categories	PDHS 2012–2013 (*n* = 2917)	PDHS 2017–2018 (*n* = 3957)	Total (*N* = 6874)
Malnutrition factors				
Coexisting forms of malnutrition	Normal	45.3%	54.0%	50.3%
	Stunting	18.1%	18.4%	18.3%
	Wasting	2.8%	2.1%	2.4%
	Underweight	1.4%	0.9%	1.1%
	Overweight/obesity	2.1%	1.6%	1.8%
	CUS	17.4%	15.3%	16.2%
	CUW	2.9%	3.0%	3.0%
	CUWS	4.6%	2.9%	3.6%
	CSO	5.1%	1.8%	3.2%
Child factors				
Had fever	Yes	39.1%	38.0%	38.5%
	No	60.9%	62.0%	61.5%
Had diarrhea recently	Yes	21.9%	19.4%	20.4%
	No	78.1%	80.6%	79.6%
Size of child at birth	Small‐sized	17.8%	16.6%	17.1%
	Average‐sized or Large‐sized	82.2%	83.4%	82.9%
Birth weight in kilograms	Normal weight	76.0%	81.9%	79.4%
	Low birth weight	23.8%	18.1%	20.5%
Birth order number	First child	21.9%	24.4%	23.3%
	Second or subsequent child	78.1%	75.6%	76.7%
Child is a twin	Single	98.1%	98.0%	98.1%
	Twin/triplet	1.9%	2.0%	1.9%
Sex of child	Male	50.4%	50.6%	50.5%
	Female	49.6%	49.4%	49.5%
Current age of child	0–11 months	18.3%	19.4%	18.9%
	12–23 months	17.7%	20.0%	19.0%
	24–35 months	21.5%	19.7%	20.5%
	36–47 months	20.8%	20.5%	20.6%
	48–59 months	21.6%	20.4%	20.9%
Maternal factors				
Maternal age	15–19 years	2.4%	2.9%	2.7%
	20–24 years	20.3%	20.4%	20.4%
	25–29 years	28.9%	31.4%	30.4%
	30–34 years	25.7%	25.5%	25.6%
	35–39 years	15.6%	14.2%	14.8%
	40–44 years	5.1%	4.2%	4.6%
	45–49 years	1.9%	1.5%	1.6%
Maternal education	No education	53.0%	51.8%	52.3%
	Primary	16.1%	13.6%	14.7%
	Secondary	20.0%	20.2%	20.1%
	Higher	10.9%	14.3%	12.9%
Working mothers	No	79.2%	88.5%	84.6%
	Yes	20.8%	11.5%	15.4%
Birth in the last 5 years	Single birth	34.1%	37.5%	36.1%
	Multiple births	65.9%	62.5%	63.9%
Smokes cigarettes	No	99.1%	96.6%	97.7%
	Yes	0.9%	3.4%	2.3%
Paternal factors				
Husband/partner′s education level	No education	30.0%	27.8%	28.7%
	Primary	14.9%	14.8%	14.8%
	Secondary	34.0%	33.9%	34.0%
	Higher	21.1%	23.5%	22.5%
Husband/partner′s occupation	Unemployed	2.5%	3.4%	3.0%
	Employed	97.5%	96.6%	97.0%
Household factors				
Type of place of residence	Rural	57.3%	54.9%	55.9%
	Urban	42.7%	45.1%	44.1%
Wealth index	Poorest	21.4%	21.2%	21.3%
	Poorer	19.9%	25.3%	23.0%
	Middle	17.8%	19.1%	18.5%
	Richer	21.5%	17.6%	19.2%
	Richest	19.5%	16.8%	18.0%
Family size	Small (1–5)	9.8%	9.5%	9.6%
	Medium (6–10)	54.7%	51.4%	52.8%
	Large (10+)	35.4%	39.2%	37.6%

## 3. Results

### 3.1. Characteristics of the Study Sample

A total of 24,471 households were sampled in this study from the PDHS (11,763 from 2012 to 2013~12,708 from 2017 to 2018). Among 24,471 households, only 8456 households had eligible children (3957 from 2012 to 2013 and 4499 from 2017 to 2018). Furthermore, data from non‐de jure residents and children with incomplete or missing anthropometry was also excluded. This resulted in a final analytical sample of 6874 children. The comparison between the PDHS 2012–2013 and PDHS 2017–2018 surveys highlights notable changes in child health, maternal and paternal characteristics, and household factors. Compared to the 2012–2013 PDHS, the recent DHS conducted in 2017–2018 showed a decrease in the prevalence of low birth weight and small birth size among children. Similarly, the 2017–2018 survey showed a lower prevalence of febrile illness and pediatric diarrhea compared with the 2012–2013 survey. However, the sex distribution almost remained stable across the two survey periods.

Between the two survey periods, several important trends emerged in maternal and paternal characteristics. Most notably, the proportion of working mothers fell sharply from 20.8% to 11.5%, whereas maternal smoking nearly quadrupled from 0.9% to 3.4%; both trends have direct implications for child health. Despite over half of mothers remaining uneducated (> 50% in both surveys), higher maternal education increased modestly. On the paternal side, educational attainment improved slightly, with fathers holding higher education rising from 21.1% to 23.5% and the share with no education declining from 30.0% to 27.8%. Full distributional details are provided in Table 2.

The urban–rural distribution remained stable, with around 55.9% of the population living in rural areas and 44.1% in urban areas. The wealth index distribution changed, with the proportion of poorer households increasing from 19.9% to 25.3%, whereas the richest category decreased from 19.5% to 16.8%. Household size remained consistent, with more than half of the families having six to 10 members (Table 3).

**Table 3:** Assessing the association of adjusted odds of water facilities with the various forms of coexisting forms of malnutrition among the infants and young children of Pakistan.

Variables	Categories	CUW^a^	CUS^b^	CUWS^c^	CSO^d^
		95% CI	95% CI	95% CI	95% CI
Child factors					
Had fever	Yes				Ref
	No				0.61 [0.43–0.85] ^∗^
Had diarrhea recently	Yes				Ref
	No				0.57 [0.36–0.90] ^∗^
Size of child at birth	Small‐sized				
	Normal‐sized				
	Large‐sized				
Birth weight in kilograms	Normal weight (2.5 kg or more)				
	Low birth weight (less than 2.5 kg)				
Birth order number					
Child is a twin	Single				
	Twin/triplet				
Sex of child	Male				
	Female				
Current age of child	0–11 months		Ref	Ref	
	12–23 months		6.35 [2.77–14.55] ^∗^	8.76 [3.52–21.77] ^∗^	
	24–35 months		10.70 [4.69–24.39] ^∗^	5.04 [1.97–12.91] ^∗^	
	36–47 months		7.12 [3.46–14.64] ^∗^	5.32 [2.26–12.53] ^∗^	
	48–59 months		6.41 [3.23–12.71] ^∗^	2.87 [1.25–6.58] ^∗^	
Maternal factors					
Maternal age	15–19 years				Ref
	20–24 years				0.28 [0.11–0.67] ^∗^
	25–29 years				0.28 [0.11–0.63] ^∗^
	30–34 years				0.28 [0.12–0.67] ^∗^
	35–39 years				0.25 [0.10–0.61] ^∗^
	40–44 years				0.67 [0.25–1.79]
	45–49 years				0.12 [0.02–0.68] ^∗^
Maternal education	No education		Ref		
	Primary		0.71 [0.35–1.41]		
	Secondary		0.36 [0.18–0.71] ^∗^		
	Higher		0.28 [0.11–0.71] ^∗^		
Working mothers	No				Ref
	Yes				0.46 [0.28–0.77] ^∗^
Birth in the last 5 years	Single birth				
	Two births				
	More than two				
Smokes cigarettes	No				
	Yes				
Paternal factors					
Husband/partner′s education level	No education	Ref	Ref		
	Primary	0.68 [0.26–1.73]	0.99 [0.42–2.35]		
	Secondary	0.33 [0.16–0.67] ^∗^	0.43 [0.22–0.83] ^∗^		
	Higher	0.89 [0.41–1.98]	0.81 [0.34–1.88]		
Husband/partner′s occupation	Unemployed				
	Employed				
Environmental factors					
Source of drinking water	Unimproved	Ref	Ref	Ref	Ref
	Improved	0.15 [0.03–0.67] ^∗^	0.36 [0.11–1.21]	0.44 [0.12–1.51]	0.41 [0.26–0.64] ^∗^
Household factors					
Type of place of residence	Rural				
	Urban				
Wealth index	Poorest			Ref	Ref
	Poorer			0.70 [0.29–1.66]	0.837 [0.52–1.33]
	Middle			0.24 [0.09–0.59] ^∗^	0.60 [0.36–1.02]
	Richer			0.48 [0.19–1.21]	1.05 [0.57–1.60]
	Richest			0.13 [0.05–0.34] ^∗^	2.43 [1.46–4.03] ^∗^
Family size	Small				
	Medium				
	Large				

*Note:* Asterisks ( ^∗^) indicate a significant association of the outcome variable.

Abbreviations: CSO, coexistence of stunting with overweight/obesity; CUS, coexistence of underweight with stunting; CUW, coexistence of underweight with wasting; CUWS, coexistence of underweight with both wasting and stunting.

^a^The odds of the coexistence of underweight with wasting were adjusted for husband education.

^b^The odds of the coexistence of underweight with stunting were adjusted for child age, maternal education, and husband education.

^c^The odds of the coexistence of underweight with both wasting and stunting were adjusted for child age and wealth index.

^d^The odds of the coexistence of stunting with overweight/obesity were adjusted for pediatric fever, pediatric diarrhea, maternal age, maternal work, and wealth index.

### 3.2. Access to Safe Drinking Water and Improved Sanitation Facilities in Pakistan Across Two Survey Periods

**Figure 2:**
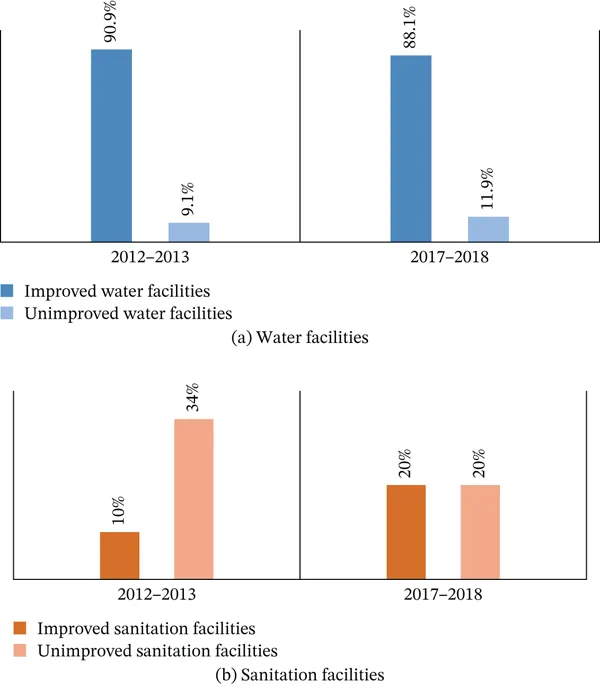
(a,b) Proportion of improved and unimproved water and sanitation facilities in Pakistan across two survey periods.

A concerning divergence emerged between water and sanitation trends across the two survey periods: access to improved toilet facilities increased from 73.7% to 79.9% (Figure 2b), reflecting gains in sanitation infrastructure, and access to safe drinking water, paradoxically declining from 90.9% in 2012–2013 to 88.1% in 2017–2018 (Figure 2a). This disconnect suggests that sanitation improvements have not been matched by equivalent progress in water quality and highlights drinking water access as a priority area for WASH policy in Pakistan.

### 3.3. Nutritional Status Profile of Children of Pakistan Across Two Survey Periods

**Figure 3:**
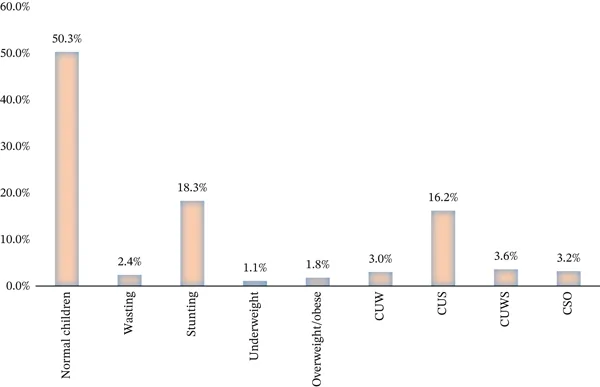
Prevalence of coexisting forms of malnutrition among under 5 children of Pakistan.

Figure 3 shows the nutritional status profile of children aged 0–59 months. The most policy‐relevant finding is that stunting is by far the dominant form of malnutrition, affecting 18.3% of normal‐weight children and 16.2% of underweight children. Critically, CFM accounted for 26% of all children sampled, whereas standalone malnutrition affected a further 23.7%. All other forms such as wasting, underweight, overweight/obesity, and their combinations individually remained below 5%, underscoring that interventions to reduce stunting should be the primary nutritional priority.

### 3.4. Assessing the Association of Source of Drinking Water With Various Forms of CFM

Table 2 showed a significant association of sources of drinking water with various forms of CFM. Provision of safe drinking water (improved water supply) significantly reduced the odds of CUW and CSO by 85% and 59%, respectively. However, no association of sources of drinking water with other forms of CFM was observed. Children without a recent history of infectious diseases such as fever and diarrhea had statistically significantly lower odds of CSO; that is, the odds of CSO were 39% lower in children not experiencing fever while 43% lower in those without diarrhea. Higher odds of CUS and CUWS were seen in older children as compared to the reference age of 0–11 months, with the greatest increase during the 2nd and 3rd years of life, implying increased nutrient requirements with growing age. Children of highly educated and working‐class women were protected against various forms of CFM. There were 72% lower odds of CUS in children of mothers with higher education as compared to no education, whereas CSO was 54% lower among children of working mothers. Increasing maternal age also had a protective association as compared to the reference age of 15–19 years. Similarly, an improvement in the wealth index showed contrasting results with various forms of malnutrition. Children from the richest households showed a decrease in the odds of CUW (OR: 0.13, CI: 0.05–0.34). Conversely, increased odds of CSO (OR: 2.43, CI: 1.46–4.03) were associated with richest household children compared to the poorest households′ children.

### 3.5. Assessing the Association of Types of Sanitation Facilities With Various Forms of CFM

Table 4 shows that the type of toilet facility had no significant effect on any type of CFM.

**Table 4:** Assessing the association of adjusted odds of sanitation facilities with the various forms of coexisting forms of malnutrition among the infants and young children of Pakistan.

Variables	Categories	CUW	CUS	CUWS	CSO
Child factors					
Had diarrhea recently	Yes				Ref
	No				0.55 [0.35–0.86] ^∗^
Size of child at birth	Small‐sized				
	Normal‐sized				
	Large‐sized				
Birth weight in kilograms	Normal weight			Ref	
	Low birth weight			0.23 [0.06–0.81] ^∗^	
Birth order number					
Child is a twin	Single				
	Twin/triplet				
Sex of child	Male				
	Female				
Current age of child	0–11 months				
	12–23 months				
	24–35 months				
	36–47 months				
	48–59 months				
Maternal factors					
Maternal age	15–19 years				Ref
	20–24 years				0.28 [0.11–0.67] ^∗^
	25–29 years				0.28 [0.11–0.63] ^∗^
	30–34 years				0.28 [0.12–0.67] ^∗^
	35–39 years				0.25 [0.11–0.61] ^∗^
	40–44 years				0.67 [0.25–1.79]
	45–49 years				0.12 [0.02–0.68] ^∗^
Maternal education	No education				
	Primary				
	Secondary				
	Higher				
Working mothers	No				0.43 [0.26–0.71] ^∗^
	Yes				Ref
Birth in the last 5 years	Single birth				
	Two births				
	More than two				
Smokes cigarettes	No				
	Yes				
Paternal factors					
Husband/partner′s education level	No education	Ref			
	Primary	0.89 [0.34–2.30]			
	Secondary	0.44 [0.21–0.92] ^∗^			
	Higher	1.40 [0.58–3.36]			
Husband/partner′s occupation	Unemployed				
	Employed				
Environmental factors					
Type of toilet facility	Unimproved	Ref	Ref	Ref	Ref
	Improved	0.79 [0.34–1.85]	0.29 [0.03–2.53]	0.74 [0.13–4.29]	1.01 [0.66–1.52]
Household factors					
Type of place of residence	Rural				
	Urban				
Wealth index	Poorest	Ref			Ref
	Poorer	1.21 [0.46–3.19]			0.83 [0.52–1.33]
	Middle	0.55 [0.20–1.52]			0.60 [0.36–1.02]
	Richer	0.45 [0.15–1.38]			1.05 [0.57–1.61]
	Richest	0.31 [0.11–0.92] ^∗^			2.43 [1.46–4.03] ^∗^
Family size	Small (1–5)				
	Medium (6–10)				
	Large (10+)				

*Note:* The asterisk ( ^∗^) denotes significant association between the study covariates and outcome.

Abbreviations: CSO, coexistence of stunting with overweight/obesity; CUS, coexistence of underweight with stunting; CUW, coexistence of underweight with wasting; CUWS, coexistence of underweight with both wasting and stunting.

Despite the absence of sanitation effects, several covariates remained significant. Children without recent fever had 38% lower odds of CSO than those without recent diarrhea (45%). Maternal age retained its protective effect on CSO across all age groups above 15–19 years. Children of working mothers had 57% lower odds of CSO, reinforcing the finding from Table 2 that maternal employment is a consistent protective factor. Paternal secondary education was associated with reduced odds of CUW compared to no education (OR: 0.44, 95% CI: 0.21–0.92). As with Table 2, the wealth index again showed opposing effects: richest households had lower odds of CUW (OR: 0.31, 95% CI: 0.11–0.92) but higher odds of CSO (OR: 2.43, 95% CI: 1.46–4.03), a robust finding across both sanitation and water models that warrants attention in nutritional policy targeting.

Thus, summarizing the results of three overarching findings emerge. First, improved water supply was the strongest environmental determinant, as it was associated with reduced odds of CUW by 85% and CSO by 59%. No significant association was found with CUS or CUWS. Second, maternal and socioeconomic factors played consistent protective roles. Children of mothers with higher education had 72% lower odds of CUS compared to those with no education, and children of working mothers had 54% lower odds of CSO. Increasing maternal age was also independently associated with CSO outcomes. Third, the wealth index showed a striking and policy‐relevant contrast. Children from the richest households had markedly lower odds of CUW (OR: 0.13, 95% CI: 0.05–0.34), yet paradoxically higher odds of CSO (OR: 2.43, 95% CI: 1.46–4.03), suggesting that rising household wealth may shift malnutrition burden from undernutrition toward CSO. Child age was also a significant predictor of CUS and CUWS, with odds peaking in the 24–35‐month age group.

## 4. Discussion

**Figure 4:**
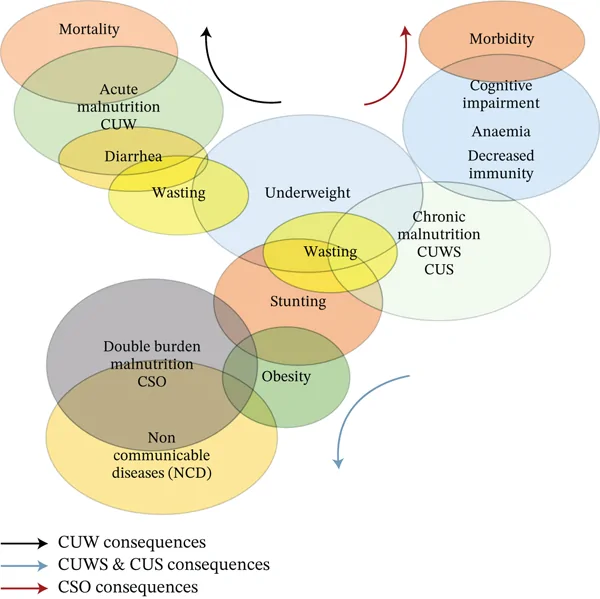
Various consequences of coexisting forms of malnutrition. This figure explains the consequences of CFM; the black arrow shows how CUW will culminate in acute malnutrition if left untreated and increase mortality; the red arrow shows how CUWS and CUS, if left untreated, are vulnerable to chronic malnutrition leading to morbidity; and the blue arrow shows how CSO, which is the double burden of malnutrition, is prone to various noncommunicable diseases later in life.

The source of drinking water is a critical determinant of child nutrition, particularly in relation to CFM. In our study, children who had access to improved water sources had 85% lower odds of CUW compared to those with unimproved water sources, indicating a strong protective association. This result is in accordance with a regional study that suggested that up to 36.5% of the underweight burden is preventable through improved water practices [37]. The robustness of this finding was due to controlling for potential confounding variables such as maternal education, household socioeconomic status, and the presence of recent illness in the child (fever and diarrhea), suggesting that the association observed between water quality and CUW is independent and meaningful in this context. The biological mechanisms linking water quality to CUW center primarily on repeated enteric infections. Fecal–oral contamination drives diarrheal disease, which in turn impairs macronutrient and micronutrient absorption, causing acute weight loss and wasting, the hallmarks of CUW. As illustrated in Figure 4 (black arrow), untreated CUW can rapidly progress to moderate and severe acute malnutrition (MAM/SAM), conditions associated with high child mortality and requiring hospitalization [38–41]. This acute trajectory explains why water quality shows the strongest and most direct protective association against CUW specifically compared to the chronic forms of malnutrition (CUS and CUWS).

This distinction between acute and chronic malnutrition pathways is an important contribution of this study. The reason water quality exerts a stronger protective effect against CUW (acute) than against CUS and CUWS (chronic) lies in the directness of the biological pathway. CUW is primarily driven by a rapid infection‐to‐malabsorption cycle; thereby, contaminated water triggers diarrheal disease, which causes immediate nutrient loss and acute wasting. Improving water quality is associated with interrupting this cycle. In contrast, CUS and CUWS develop over months and years through cumulative and multifactorial processes, including chronic dietary inadequacy, repeated subclinical infections, poor maternal nutrition, and household‐level socioeconomic deprivation, none of which are resolved by water quality improvements alone. Improved water supply breaks the infection–malabsorption cycle that drives acute wasting and underweight (CUW), but its protective effect against CUS and CUWS, the chronic forms, is weaker and nonsignificant. This is consistent with the understanding that chronic malnutrition (stunting‐related) accumulates over months and years, driven by multifactorial influences including dietary inadequacy, recurrent subclinical infections, maternal nutrition, and socioeconomic deprivation rather than acute waterborne illness alone [42–44]. As shown in Figure 4 (red arrow), CUS and CUWS, if left unaddressed, compound into chronic morbidity with lifelong developmental consequences. Addressing these chronic forms therefore likely requires broader multisectoral interventions beyond water quality, including dietary diversity, maternal health, and socioeconomic support [31, 32].

Stunting (short stature for age), also called chronic malnutrition, is associated with developmental impairments and reduced economic potential later in life [45, 46]. The links between WASH and stunting are well established in the literature involving diarrhea, EED, and chronic undernutrition [42]. This study adds nationally representative Pakistani evidence that stunting‐related CFM (CUS and CUWS) is not significantly reduced by improved water supply alone. This finding advances the evidence base by suggesting that water access, while critical for acute malnutrition, is insufficient on its own to address the multifactorial drivers of chronic stunting. Consistent with studies in South Asia and sub‐Saharan Africa [45, 46], our results indicate that reducing CUS and CUWS requires complementary interventions, improved dietary adequacy, food supplementation, maternal nutrition, and broader socioeconomic support [44, 47].

Our study found a statistically significant association between improved drinking water sources and lower odds of CSO (see Table 2) among children under 5 in Pakistan. Specifically, children from households with improved water sources had 59% lower odds of experiencing CSO compared to those with unimproved water sources. As illustrated in Figure 4 (blue arrow), CSO represents the DBM, with long‐term consequences for noncommunicable disease (NCD) risk such as diabetes, dyslipidemia, and cardiovascular diseases [48, 49]. The biological mechanisms underlying CSO are complex. Households facing water insecurity may resort to cheaper, energy‐dense but nutrient‐poor foods, producing simultaneous stunting and excess adiposity [9]. The complex interplay between early undernutrition and later overnutrition also exacerbates NCD risk, a trend rising rapidly in LMICs. It has been hypothesized that environmental pollutants, including endocrine‐disrupting chemicals (EDCs) such as bisphenol A and phthalates present in contaminated water, may have obesogenic properties [48, 49]. However, this study did not measure EDC exposure directly, and this mechanism should be treated as a speculative pathway requiring dedicated future investigation, not a finding of the present study. Urbanization and the growing availability of ultraprocessed foods in Asia have also been implicated in the rising prevalence of CSO [50, 51].

Despite the nationally representative dataset of our country, consistent sample size, and multistage stratified cluster sampling, this study found no significant association between the sanitation facility (improved vs. unimproved) and various forms of CFM among children under 5 years of age. This null finding, however, is itself informative. The DHS sanitation variable captures only the type of toilet facility available at the household level and does not account for the actual cleanliness and maintenance, thereby failing to detect an association with coexisting malnutrition in this study [52]. Taken together, these findings suggest that facility‐type improvements must be combined with hygiene education, water quality improvements, and environmental sanitation to effectively reduce malnutrition. This is consistent with evidence from eight LMICs showing sanitation can prevent stunting [53].

Our study found that children who lived in households with improved water conditions are significantly less likely to experience CSO, with a 38%–39% chance if there are no recent illnesses like fever and a 43%–45% lower chance if there is no history of recent diarrhea, compared to children who have a history of recent illness, a history of fever, and diarrhea. A study conducted by Khaliq et al. also revealed similar results for nutritional paradox [54]. This is in accordance with the finding that obesity impairs immunity, leading to frequent infections and perpetuating a vicious cycle of infection and malnutrition, and in turn complicates weight management [55]. The lack of significant association between fever, diarrhea, and other forms of malnutrition in our study indicates that the relationship between WASH and CFM is complex, involving environment, genetics, and nutritional factors beyond infection alone.

A higher likelihood of CUS and CUWS was observed in older children compared to infants aged 0–11 months. This is supported by several regional and global studies. Research from Gambia, Malawi, and Ethiopia shows that children aged 12–23 months are more likely to experience both stunting and underweight than those aged 0–11 months [56]. Another study found that children aged 9–23 months had a higher risk of CUS and CUW compared to younger infants [57]. This trend is attributed to the accumulation of nutritional deficits over time, higher nutrient demands as children grow, and increased exposure to infections and suboptimal feeding practices during the weaning period [56, 58]. Older children may face greater risks of malnutrition if complementary feeding is inadequate, dietary diversity is poor, or if there are gaps in continued nutritional and health support [58, 59].

Among maternal factors, increasing maternal age, particularly beyond the reference age of 15–19 years, was found to have protective effects against pediatric CFM. Studies from South Asia and Africa consistently show that children born to younger mothers, especially under 19 or 20 years, are at significantly higher risk of stunting, wasting, and underweight compared to those born to older mothers. For example, in Bangladesh, children of mothers under 19 were 1.4–1.6 times more likely to be malnourished than those of older mothers [60]. This pattern is echoed in systematic reviews covering Pakistan, Ethiopia, Ghana, and other countries, which found that the risk of stunting drops as maternal age increases [60, 61]. Similarly, children of working women had lower chances of CSO (54%–57%) compared to unemployed women.

Employment may provide better financial resources for food and healthcare, as well as improved knowledge about nutrition and health practices, positively influencing child health. Working mothers may also be more engaged in community health programs, especially in areas with improved sanitation, which can further enhance the well‐being of their children [62].

Both maternal and paternal education have a significant role in reducing the odds of CUS and CUW, as evident from our study compared to children with uneducated mothers and fathers. Studies from South Asia, including Pakistan [63], consistently show that higher education is associated with lower rates of various forms of malnutrition among children. Educated parents, particularly mothers, are more likely to understand the importance of hygiene, sanitation, and proper nutrition, leading to better child health outcomes [64]. Educated fathers are more likely to contribute to better household income, prioritize health and nutrition, and support improved hygiene and sanitation practices, all of which protect against child malnutrition [65]. Some large multicountry analyses confirm that both maternal and paternal education independently reduce childhood undernutrition [66].

A particularly noteworthy and original contribution of this study is the contrasting effect of household wealth on different forms of CFM, a finding that reflects emerging nutritional transition dynamics in Pakistan. These contrasting wealth effects, detailed in Table 2, suggest that higher household wealth does not uniformly improve nutritional outcomes [67]. While wealth is associated with protection against acute undernutrition (CUW), it is linked with greater odds of the double‐burden phenotype (CSO), possibly reflecting dietary transitions toward energy‐dense but nutrient‐poor foods as income rises.

However, the association of wealth with stunting alongside obesity indicates that merely increasing wealth without focusing on health equity and nutrition can lead to complex malnutrition issues [68]. In LMICs, undernutrition is consistently more prevalent among the poorest households, who lack access to adequate food, healthcare, and sanitation [69]. However, as wealth increases, especially in urban and transitioning economies, there is a growing trend of overnutrition and the DBM, where stunting coexists with obesity or overweight in the same child or household [51]. Yet, the economic category of a country alone does not fully explain these disparities—local context, health systems, and social norms also play roles [70]. The paradox observed in our study increased stunting with obesity among the richest—mirrors a global trend where economic growth and urbanization lead to greater consumption of energy‐rich but nutrient‐poor foods, resulting in simultaneous under‐ and overnutrition within populations and even individuals [71]. These patterns highlight that simply increasing wealth or improving infrastructure is not enough; targeted nutrition education, food quality improvement, and health equity measures are essential to address both under‐ and overnutrition [70].

### 4.1. Strengths and Limitations

This study provides a deep and clear understanding of WASH and CFM among children under 5 years by utilizing the most recent dataset of the last two waves of PDHS conducted in 2012–2013 and 2017–2018. The findings of this study are nationally representative because the PDHS is a nationally representative dataset that collects information related to the health, nutrition, demography, and household features of residents living in the urban and rural communities of the country. Furthermore, the multistage stratified cluster sampling methods further enhance the sample representativeness of this study. The data collection method, including the assessment methods for each exposure WASH and outcome CFM of this study, was carried out by a team of trained and experienced data collectors using validated and reliable sets of questionnaires. The exposure variable of this study, that is, WASH, has various categories, and these categories were derived from the core questions and indicators for monitoring WASH in healthcare facility guidelines. Similarly, the anthropometry of each child was done using the standardized weighing scale and stadiometer by a team of anthropometrists. Moreover, the exclusion of missing anthropometric values and anthropometric outliers further enhances the robustness of this study′s findings. Despite several study strengths, the cross‐sectional study design limits the ability to develop causal relationships between WASH conditions and CFM. Second, dietary diversity, a key mediator of malnutrition, was not captured in this dataset, representing a source of potential residual confounding that may have attenuated or inflated some associations. Third, several child health variables (fever and diarrhea) relied on maternal self‐report, introducing recall bias, as mothers may not accurately remember illness episodes within the reference period. Because exposure (WASH) and outcome (CFM) were measured simultaneously, no temporal sequencing can be established; it is not possible to determine whether poor WASH preceded malnutrition or vice versa. Moreover, the responses of the mothers for certain variables cannot be validated because of the risk of social desirability and response bias. Thereby, when making any judgment with the findings of this study, one must need to consider the elements of social desirability bias and reporting bias.

### 4.2. Future Research Direction

To enhance the understanding between WASH conditions and CFM among children under 5 years of age in Pakistan, future research should engage in longitudinal studies with repeated observations over an extended period to further investigate the causal relationship between WASH variables and CFM that were not fully captured through this cross‐sectional study design.

Researchers should also focus on long‐term cohort studies tracking children from birth to school age, with particular attention to those from poor households and areas with unimproved sanitation. Additionally, future studies should explore other underlying factors beyond WASH that contribute to malnutrition, including nutritional status, food intake, and dietary habits in children from low‐socioeconomic backgrounds.

### 4.3. Implications for Public Health

The association between WASH conditions and the prevalence of CFM among children under 5 years of age in Pakistan has serious public health implications. Proper interventions and campaigns to educate people, such as enhancing access to clean water, improving sanitation facilities, and promoting hand hygiene, can significantly reduce malnutrition rates. Public health programs should focus on addressing the indirect links through community awareness campaigns, which are crucial for breaking the cycle of malnutrition, alongside fostering collaborations between government and nongovernmental organizations contributing toward the achievement of SDGs related to health, nutrition, and water and sanitation access.

## 5. Conclusions

The findings of this study underscore the critical importance of household WASH conditions in the context of child malnutrition in Pakistan. To the best of our knowledge, this is the first nationally representative study in Pakistan to examine the relationship between WASH conditions and CFM, providing novel evidence to guide targeted public health interventions. Improved access to safe drinking water was significantly associated with reductions in coexisting malnutrition. Specifically, CUW and CSO showed notable decreases in odds among children from households with improved water supply. Beyond these direct associations, maternal education, employment status, and household socioeconomic conditions emerged as key indirect determinants of CFM, underscoring the need for integrated approaches that address both WASH infrastructure and its broader social determinants. These findings call for a comprehensive, multisectoral response, specifically, the integration of WASH programs with maternal education initiatives, targeted nutrition counseling campaigns for households with young children, and nutrition‐sensitive social protection schemes that simultaneously address water access and food security. While the cross‐sectional design of this study limits causal inference and self‐reported data introduce potential recall bias, these findings provide a robust, nationally representative foundation for policy prioritization. Future longitudinal research should build on this evidence to establish causal pathways and test the effectiveness of integrated WASH‐nutrition interventions in Pakistan.

**Nomenclature**
AIC: Akaike information criteria
AJK: Azad Jammu and Kashmir
BIC: Bayesian information criteria
CFM: coexisting forms of malnutrition
CI: confidence interval
CSO: coexistence of stunting with overweight/obesity
CUS: coexistence of underweight with stunting
CUW: coexistence of underweight with wasting
CUWS: coexistence of underweight, wasting, and stunting
EBs: enumeration blocks
EDC: endocrine‐disrupting chemicals
EED: environmental enteric dysfunction
FATA: Federally Administered Tribal Areas
FTIs: fecal‐transmitted infections
GB: Gilgit Baltistan
ICT: Islamabad Capital Territory
LAZ/HAZ: length/height for age *z*‐score
LMICs: lower‐ and middle‐income countries
MAM: moderate acute malnutrition
MRM: micronutrient‐related malnutrition
NIPS: National Institute of Population Studies
NNS: National Nutrition Survey
NTDs: neglected tropical diseases
OR: odds ratio
PBS: Pakistan Bureau of Statistics
PDHS: Pakistan Demographic and Health Survey
PPS: probability proportional to size
PSUs: primary sampling units
SAM: severe acute malnutrition
SDGs: Sustainable Development Goals
SFM: standalone forms of malnutrition
STH: soil‐transmitted helminths
UNICEF: United Nations International Children′s Emergency Fund
VIFs: variance inflation factors
WASH: water, sanitation, and hygiene
WAZ: weight for age *z*‐score
WHO: World Health Organization
WHZ: weight for length/height *z*‐score

## Author Contributions

B.A.: original draft writing, visualization, and formatting of the manuscript. H.A.R.A.: data cleaning, result writing, and assisted A.K. in data analysis. F.S.: assisted B.A. in writing the original draft of the manuscript. I.A.U.: developed the initial draft of the manuscript, crafted the research proposal, and helped in data analysis. M.H.K.: worked on methodology and participated in reviewing and editing the original draft. K.A.K.: participated in critical reviewing and editing of the manuscript. A.K.: conceptualization, data management, data analysis, reviewing, editing, and supervision.

## Funding

Open access publishing facilitated by Queensland University of Technology (10.13039/501100001793), as part of the Wiley—Queensland University of Technology agreement via the Council of Australasian University Librarians

## Disclosure

All authors reviewed and approved the final draft of the manuscript. In this research, the DHS data of Pakistan was used. To access this data, a formal application was submitted, which included our intention to publish the findings. The DHS data archivist provided us with a letter granting permission to publish our research, with the condition that we acknowledge the DHS data repository in the acknowledgments section of the publication.

## Ethics Statement

In this study, an ethical approval waiver was granted because the data used was deidentified, and the research team had no direct contact with the study population. Consequently, individual participant consent and ethical approval of this study were not required. However, the data in each DHS was collected following the ethical principles of Helsinki′s Declaration.

## Consent

In this study, the research team utilized deidentified data obtained from the DHS repository. This data does not contain any information that could be used to identify or trace individual participants. Consequently, individual participant consent was not required by the primary research team for this study.

## Conflicts of Interest

The authors declare no conflicts of interest.

## Supporting information


**Supporting Information** Additional supporting information can be found online in the Supporting Information section. File S1: The supporting information has three tables, which present nutritional outcomes and their coding, unadjusted odds of water and sanitation facilities with the various forms of coexisting forms of malnutrition, and a *z*‐score anthropometry chart.

## Data Availability

The data that support the findings of this study are openly available in the DHS Program at https://dhsprogram.com/.
