# Supplementary material for: Exploring Beyond the Cyclical Dynamics of Water, Sanitation, and Hygiene With Coexisting Forms of Malnutrition Among Children Under 5 in Pakistan
Source: Int J Pediatr. 2026 Sep 9;2026:6378814. doi: 10.1155/ijpe/6378814 (PMC13555817; doi:10.1155/ijpe/6378814)
Supplement: Supplementary file 1 [file IJPE-2026-6378814-s001.docx]

**SUPPLEMENTARY TABLE 1: Nutritional outcomes and their coding.**

| Numeric code | Nutritional outcome |
| --- | --- |
| 00 | Normal |
| 1.00 | Stunting |
| 2.00 | Wasting |
| 5.00 | Underweight |
| 6.00 | Coexistence of underweight with stunting |
| 7.00 | Coexistence of underweight and wasting |
| 8.00 | Coexistence of underweight with wasting and stunting |
| 10.00 | Overweight/obesity |
| 11.00 | Coexistence of stunting with overweight and obesity |

**SUPPLEMENTARY TABLE 2: Assessing the association of unadjusted odds water and sanitation facilities with the various forms of coexisting forms of malnutrition among the infants and young children of Pakistan.**

| Variables | Categories | CUW | CUS | CUWS | CSO |
| --- | --- | --- | --- | --- | --- |
| Year | 2017-2018 | Ref | Ref | Ref | Ref |
|  | 2012-2013 | 0.59 (0.34-1.01) | 0.71 (0.44-1.12) | 0.97 (0.58-1.62) | 0.34 (0.25-0.46) * |
| Child factors | | | | | |
| Had fever | No | Ref | Ref | Ref | Ref |
|  | Yes | 0.64 (0.37-1.09) | 0.56 (0.35-0.89) * | 0.62 (0.37-1.04) | 0.56 (0.41-0.77) * |
| Had diarrhea recently | No | Ref | Ref | Ref | Ref |
|  | Yes | 1.15 (0.62-2.10) | 0.89 (0.52-1.52) | 0.94 (0.52-1.72) | 0.54 (0.35-0.84) * |
| Size of child at birth | Average sized or  Large sized | Ref | Ref | Ref | Ref |
|  | Small sized | 0.51 (0.26-0.98) * | 0.94 (0.55-1.62) | 0.97 (0.53-1.76) | 1.08 (0.74-1.58) |
| Birth weight in kilograms | Low birthweight | Ref | Ref | Ref | Ref |
|  | Normal weight | 1.61 (0.46-5.67) | 1.09 (0.39-3.01) | 0.23 (0.06-0.82) * | 2.28 (0.89-5.85) |
| Birth order number | First childbirth | Ref | Ref | Ref | Ref |
|  | Subsequent birth | 0.93 (0.48-1.81) | 0.91 (0.51-1.63) | 0.87 (0.46-1.66) | 0.96 (0.68-1.37) |
| Child is twin | Single | Ref | Ref | Ref | Ref |
|  | Twin/Triple | 0.28 (0.07-1.10) | 0.33 (0.12-0.89) * | 0.35 (0.11-1.20) | 0.74 (0.22-2.51) |
| Sex of child | Male | Ref | Ref | Ref | Ref |
|  | Female | 1.01 (0.59-1.69) | 0.91 (0.57-1.45) | 0.63 (0.37-1.06) | 0.98 (0.73-1.31) |
| Current age of child | 0-11 months | Ref | Ref | Ref | Ref |
|  | 12- 23 months | 2.14 (0.92-4.91) | 5.16 (2.39-11.17) * | 7.26 (3.15-16.69) * | 0.12 (0.06-0.21) * |
|  | 24-35 months | 2.34 (0.98-5.56) | 9.77 (4.38-21.74) * | 5.37 (2.27-12.94) * | 0.18 (0.11-0.29) * |
|  | 36-47 months | 1.23 (0.56-2.70) | 6.40 (3.20-12.79) * | 3.80 (1.74-8.30) * | 0.20 (0.12-0.33) * |
|  | 48-59 months | 1.02 (0.47-2.18) | 5.71 (2.96-11.02) * | 2.49 (1.15-5.36) * | 0.26 (0.16-0.41) * |
| Maternal factors | | | | | |
| Maternal age | 15-19 years | Ref | Ref | Ref | Ref |
|  | 20-24 years | 2.01 (0.56-7.14) | 3.33 (1.11-9.97) * | 1.72 (0.51-5.77) | 0.33 (0.14-0.76) * |
|  | 25-29 years | 1.04 (0.31-3.48) | 1.93 (0.69-5.39) | 1.12 (0.35-3.50) | 0.35 (0.16-0.77) * |
|  | 30-34 years | 2.10 (0.61-7.25) | 3.09 (1.06-9.04) * | 1.63 (0.49-5.34) | 0.46 (0.21-1.02) |
|  | 35-39 years | 3.13 (0.75-12.93) | 4.85 (1.38-17) * | 2.27 (0.57-8.97) | 0.41 (0.18-0.93) * |
|  | 40-44 years | 1.41 (0.27-7.13) | 2.73 (0.67-10.98) | 2.04 (0.45-9.29) | 0.93 (0.37-2.35) |
|  | 45-49 years | 3.75 (0.34-41.1) | 3.89 (0.42-35.85) | 0.91 (0.06-12.52) | 0.22 (0.04-1.16) |
| Maternal education | No education | Ref | Ref | Ref | Ref |
|  | Primary | 0.45 (0.21-0.95) * | 0.49 (0.26-0.92) * | 0.28 (0.13-0.60) * | 0.99 (0.65-1.52) |
|  | Secondary | 0.46 (0.23-0.93) * | 0.31 (0.16-0.55) * | 0.37 (0.19-0.73) * | 0.96 (0.64-1.44) |
|  | Higher | 0.41 (0.16-1.02) | 0.24 (0.11-0.53) * | 0.23 (0.09-0.61) * | 1.72 (1.08-2.75)* |
| Working mothers | No | Ref | Ref | Ref | Ref |
|  | Yes | 0.65 (0.33-1.28) | 1.08 (0.61-1.90) | 0.91 (0.48-1.72) | 0.55 (0.34-0.88) * |
| Birth in last five years | Single birth | Ref | Ref | Ref | Ref |
|  | Multiple birth | 0.67 (0.38-1.19) | 0.85 (0.51-1.42) | 0.75 (0.43-1.31) | 0.78 (0.58-1.04) |
| Smokes cigarettes | No | Ref | Ref | Ref | Ref |
|  | Yes | 1.51 (0.166-13.75) | 2.18 (0.29-16.1) | 1.88 (0.22-15.83) | 1.44 (0.62-3.35) |
| Paternal factors | | | | | |
| Husband/partner's education level | No education | Ref | Ref | Ref | Ref |
|  | Primary | 0.80 (0.32-1.92) | 0.81 (0.35-1.83) | 0.75 (0.31-1.82) | 0.75 (0.46-1.23) |
|  | Secondary | 0.38 (0.19-0.74) * | 0.35 (0.19-0.64) * | 0.29 (0.15-0.55) * | 1.01 (0.71-1.46) |
|  | Higher | 0.97 (0.44-2.13) | 0.38 (0.18-0.79) * | 0.58 (0.26-1.27) | 1.61 (1.09-2.37) * |
| Husband/partner's occupation. | Unemployed | Ref | Ref | Ref | Ref |
|  | Employed | 1.01 (0.25-3.88) | 1.22 (0.36-4.06) | 1.09 (0.28-4.12) | 0.45 (0.22-0.91) * |
| Environmental factors | | | | | |
| Source of drinking water | Unimproved | Ref | Ref | Ref | Ref |
|  | Improved | 0.25(0.07-0.87)* | 0.28(0.08-0.90)* | 0.22(0.06-0.74)* | 0.45(0.31-0.66)* |
| Type of toilet facility | Unimproved | Ref | Ref | Ref | Ref |
|  | Improved | 0.90(0.47-1.72) | 0.49(0.28-0.87)* | 0.41(0.22-0.75)* | 0.97(0.69-1.35) |
| Household factors | | | | | |
| Type of place of residence | Urban | Ref | Ref | Ref | Ref |
|  | Rural | 1.39(0.823-2.35) | 2.15 (1.35-3.41) * | 1.38 (0.82-2.3) | 0.70 (0.52-0.93) * |
| Wealth index | Poorest | Ref | Ref | Ref | Ref |
|  | Poorer | 1.42 (0.62-3.26) | 0.73 (0.34-1.56) | 0.72(0.32-1.61) | 0.71 (0.46-1.05) |
|  | Middle | 0.68 (0.29-1.55) | 0.34 (0.16-0.71) * | 0.27 (0.12-0.62) * | 0.51 (0.32-0.82) * |
|  | Richer | 0.57 (0.23-1.3) | 0.42 (0.19-0.90) * | 0.45 (0.19-1.03) | 0.80 (0.51-1.25) |
|  | Richest | 0.41 (0.17-0.96) * | 0.14 (0.06- 0.29) * | 0.13 (0.05-0.32) * | 1.75 (1.14-2.69) * |
| Family size | Small | Ref | Ref | Ref | Ref |
|  | Medium | 1.57 (0.64-3.85) | 1.24 (0.58-2.6) | 1.20 (0.51-2.81) | 1.24 (0.58-2.64) |
|  | Large | 1.56 (0.62-3.89) | 1.25 (0.57-2.71) | 1.29 (0.54-3.06) | 1.25 (0.57-2.71) |

Where,

CUW = Coexistence of underweight with wasting, CUS = Coexistence of underweight with stunting, CUWS = Coexistence of underweight with both wasting and stunting, and CSO = Coexistence of stunting with overweight/obesity.

**SUPPLEMENTARY Table 3: Z-score-based classification of nutritional status in paediatric populations^22^**

| Nutritional category | WAZ | WHZ | HAZ |
| --- | --- | --- | --- |
| Normal | -1.99 to +1.99 | -1.99 to +1.99 | -1.99 to +1.99 |
| Stunting | -1.99 to +1.99 | -1.99 to +1.99 | ≤ -2.00 |
| Underweight | ≤ -2.00 | -1.99 to +1.99 | -1.99 to +1.99 |
| Wasting | -1.99 to +1.99 | ≤ -2.00 | -1.99 to +1.99 |
| Overweight/Obesity | -1.99 to +1.99 | ≥ -2.00 | -1.99 to +1.99 |
| Coexistence Underweight + Wasting | ≤ -2.00 | ≤ -2.00 | -1.99 to +1.99 |
| Coexistence Underweight + Stunting | ≤ -2.00 | -1.99 to +1.99 | ≤ -2.00 |
| Coexistence Underweight + Wasting + Stunting | ≤ -2.00 | ≤ -2.00 | ≤ -2.00 |
| Coexistence Stunting + Overweight/Obesity | -1.99 to +1.99 | ≥ -2.00 | ≤ -2.00 |

●Normal range (-1.99 to +1.99), ●Low range (≤ -2.00), ● High range (≥ -2.00).
